# Multifunctional Liquid Metal for Biomimicry Application

**DOI:** 10.3390/biomimetics10090574

**Published:** 2025-08-29

**Authors:** Yi-Ran Xu, You-Long Li, Yu-Kun Yi, Heng-Yang Bao

**Affiliations:** School of Mechanical Engineering, Anhui University of Technology, Ma’anshan 243032, China; liyoulong@ahut.edu.cn (Y.-L.L.); yiyukun040610@ahut.edu.cn (Y.-K.Y.); bhy20040307@ahut.edu.cn (H.-Y.B.)

**Keywords:** liquid metal, 3D printing, catalyze, sensing, biomedicine, flexible electronics and devices

## Abstract

Liquid metal (LM), which possesses unique material properties such as excellent flexibility, high thermal and electrical conductivities, and biocompatibility, has demonstrated broad application potential in the fields of intelligent manufacturing, flexible electronics, and biomedical engineering. This paper presents a systematic review of recent advances in multifunctional LM materials for biomimetic applications, with a focus on 3D printing, catalysis, sensing, and biomedical technologies. Through advanced 3D printing techniques—including direct writing, embedded printing, and extrusion/infiltration—LM has been effectively utilized in the fabrication of high-precision electronic components. In catalysis, LM-based catalysts exhibit superior performance in energy conversion and environmental remediation due to their high catalytic activity and selectivity. Moreover, LM has made notable progress in the development of high-performance sensors and biomedical devices, contributing significantly to the advancement of health monitoring and intelligent diagnostic and therapeutic technologies. This review aims to provide theoretical insights and technical references for further research and engineering applications of liquid metals.

## 1. Introduction

In recent years, with the rapid advancement of cutting-edge technologies such as flexible electronics [1], biomedical engineering [2], and intelligent manufacturing [3], there has been an escalating demand for materials that exhibit high electrical conductivity, excellent flexibility, biocompatibility, and functional programmability. Against this backdrop, liquid metals (LMs) have garnered widespread attention due to their unique physicochemical properties and have gradually become a core material in interdisciplinary research spanning multiple fields. Notably, LMs represented by gallium-based alloys (such as eutectic gallium-indium alloy, EGaIn) and bismuth-based alloys (such as bismuth-indium-tin alloy, BiInSn) possess metallic-level electrical conductivity, low modulus, high deformability, and exceptional self-healing capabilities [4,5]. Combining both conductivity and flexibility, these materials surpass traditional rigid conductive materials.

The uniqueness of LMs lies not only in their fluidity and malleability but also in their highly compatible interfacial behavior and surface chemical properties. For instance, EGaIn rapidly forms a self-limiting gallium oxide skin in air, which provides mechanical stability during flow, enabling patterning, circuit formation, and even three-dimensional (3D) structural construction [6,7]. This property facilitates their use not only in the development of flexible and wearable circuits, but also in microscale integration for applications such as microfluidics, biosensing, and in vivo implantation [8]. Meanwhile, LMs exhibit diverse stimulus-responsive properties, including photothermal [9], electric field [10], magnetic field [11], and chemical stimulation responses [12], conferring significant advantages in constructing intelligent responsive systems. In terms of photothermal conversion, LMs demonstrate efficient energy absorption and localized heating capabilities, finding widespread applications in photothermal therapy, temperature-controlled drug release, and tissue modulation [13,14,15]. Under electric field actuation, LMs can perform self-propelled migration, pattern reconstruction, and electronic switching [16,17]. They can also be electrochemically controlled to achieve behaviors such as rotation, levitation, and electrode-to-electrode transfer without short-circuiting [18]. These capabilities further enhance their use in reconfigurable circuits and bio-inspired fluidic systems. Furthermore, the introduction of magnetic nanoparticles into magnetically controlled LM systems has unveiled remarkable potential in emerging medical scenarios such as remote manipulation, targeted delivery, and in vivo surgical operations [19,20].

**Figure 1:**
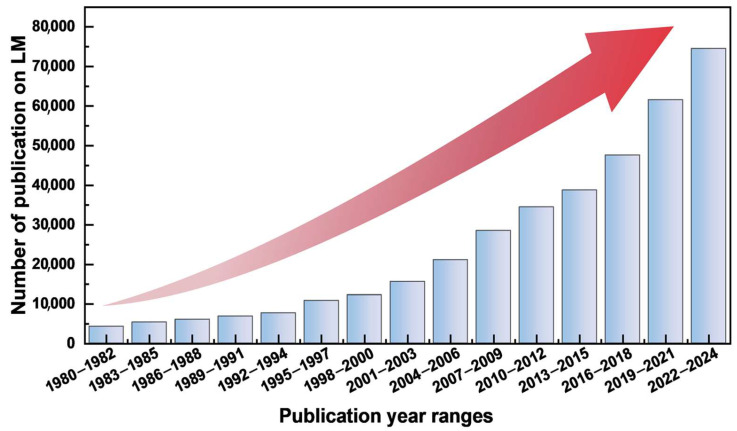
Research trends in the field of LM materials are illustrated by the number of publications per year (Data from the Web of Science).

As shown in Figure 1, research interest in LM-related materials and devices has witnessed explosive growth over the past few years, aligning with the rapid development of soft functional materials and flexible electronics. The interdisciplinary convergence of disciplines such as materials science [21], mechanical engineering [22], biomedicine [23], and electronic engineering [24] has propelled LMs research from material property characterization to device integration and system-level applications. Table 1 lists the fundamental physicochemical properties of representative LMs, which underpin their multifunctional applications across 3D printing, catalysis, sensing, and biomedical fields. These intrinsic properties provide the basis for high conductivity, processability, interfacial stability, and biocompatibility, enabling LMs to meet the demands of advanced technologies [25].

In the field of 3D printing, traditional plastic materials face challenges in meeting the manufacturing requirements of high-precision electronic components and electromagnetic shielding devices due to their limited insulation and conductivity. In contrast, LM 3D printing technology offers a novel solution for fabricating complex circuits and functional devices, leveraging its high conductivity and superior molding capabilities [28]. Notably, the emergence of direct-write printing, embedded printing, and extrusion/infiltration printing techniques has further advanced the application of LMs in micro-nano electronics, flexible sensors, and bio-integrated systems [29,30,31]. In the realm of catalytic reactions, LM catalysts outperform traditional solid catalysts by virtue of their high electrical conductivity, excellent interfacial electron transfer capabilities, and surface isotropy [32,33]. LM catalysts not only effectively enhance reaction efficiency and selectivity but also enable self-regeneration through dynamic interfacial modulation, playing a pivotal role in energy conversion, environmental remediation, and material synthesis [34,35].

The fields of flexible electronics and soft robotics have also benefited from the unique properties of LMs. By integrating LMs into elastic matrices, highly stretchable and compliant electronic devices and robotic components can be fabricated, enabling innovative applications in health monitoring, human–machine interaction, and medical exploration [36,37]. Additionally, remarkable progress has been made in the application of LMs in the biomedical field, ranging from neural signal monitoring to visual function restoration, tumor treatment, and systemic disease intervention [38,39,40]. LMs are gradually emerging as a key material for next-generation bioelectronics and intelligent therapeutic systems.

**Figure 2:**
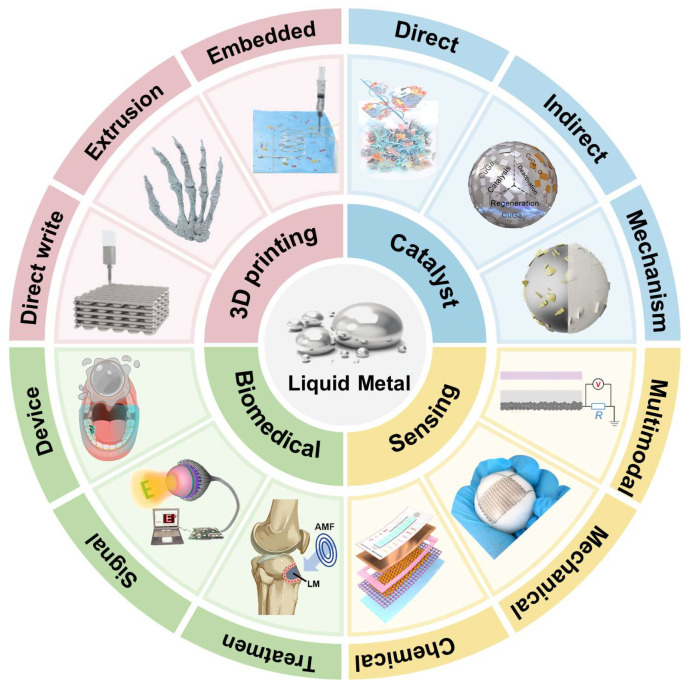
The Cutting-edge Applications of LMs in various fields.

The development of LM materials has advanced from fundamental research to application-oriented transformation. With their multi-scale, multi-physical field coupling characteristics and cross-platform integration capabilities, LMs are emerging as a bridge connecting materials science and device engineering. This paper aims to systematically review the development achievements of LMs in key research directions in recent years, with a particular focus on their representative works in critical areas such as 3D printing, catalysis, sensing, and biomedical applications. Figure 2 provides a clear and intuitive overview of the cutting-edge applications and major research progress of LMs across multiple fields. It summarizes the functional properties, integration strategies, and practical performance of LM materials, while highlighting key challenges and potential directions for future development. Through this review, we aspire to offer systematic theoretical support and technical inspiration for the in-depth research and engineering translation of LM materials.

## 2. Liquid Metal 3D Printing

Mainstream metal 3D printing methods, such as selective laser melting and electron beam melting, have demonstrated remarkable success in fabricating complex metal parts, enhancing the performance and customization capabilities of aerospace components and medical devices. However, these techniques face several challenges: the variable properties of metal powders can compromise printing precision; the high-temperature environments result in substantial energy consumption, elevated costs, and accelerated equipment degradation; and the limited range of printable materials hinders the development of multifunctional systems [41,42,43].

To address these bottlenecks, recent research has increasingly focused on low-melting-point metals, particularly the application of LMs in 3D printing. LM 3D printing offers several advantages, including rapid fabrication of lightweight, highly deformable conductive structures, low-temperature processing, high material utilization efficiency, and excellent interfacial adaptability [28]. In portable and wearable electronics, LMs have been utilized to fabricate flexible interconnects and deformable sensing layers, significantly improving system integrability and operational stability. Moreover, by integrating LMs with polymers or nanomaterials, it is possible to construct multifunctional systems combining electrical conductivity, thermal conductivity, magnetic responsiveness, and mechanical compliance [44]. For example, through formulation engineering, composite sensors based on LMs and elastomers can detect mechanical stimuli such as pressure and strain, while simultaneously capturing electrophysiological signals, thereby supporting applications in health monitoring and intelligent diagnostics [45].

**Figure 3:**
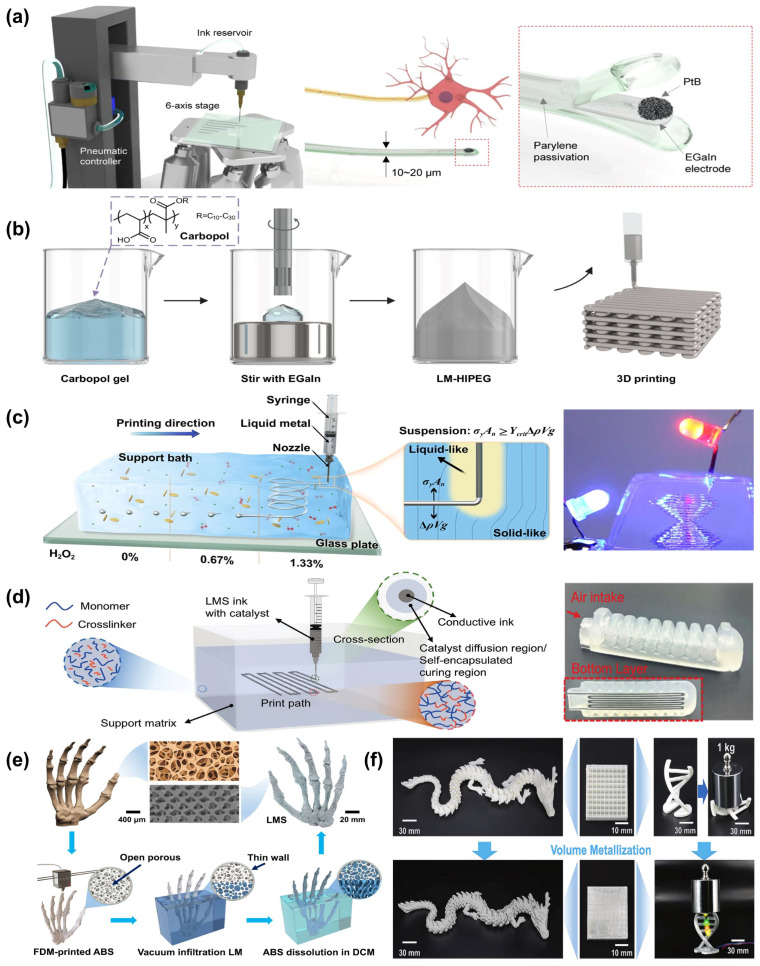
Major 3D printing technologies of LMs. Direct-Ink Writing: (**a**): LM DIW 3D printing of soft neural probes [46]; (**b**): High internal phase emulsions gel ink for DIW 3D printing of LM [47]; Embedded Printing: (**c**): Schematic of LM suspension printing in AAm/nanoclay support bath with hydrogen peroxide [48]; (**d**): Conceptual design diagram of LMS ink in the embedded 3D printing process [49]; Extrusion and Infiltration Printing: (**e**): Fabrication of 3D complex-shaped LM skeleton-based active-cooling elastomer [50]; (**f**): Schematic of VMPC preparation and characterization [44].

Given the significant research value and broad application prospects of LM 3D printing, scholars worldwide have devoted increasing efforts to its advancement. Currently, as depicted in Figure 3, major LM 3D printing techniques include direct ink writing, embedded printing, and extrusion/infiltration-based methods. This section provides a systematic introduction and in-depth discussion of the working principles, processing characteristics, and future development trends of these technologies, aiming to inform and guide further progress in the field of LM 3D printing.

### 2.1. Direct-Ink Writing of Liquid Metal

Direct ink writing (DIW) 3D printing technology enables the precise and direct deposition of low-melting-point metals onto substrate surfaces through a nozzle, using specially formulated inks to construct self-supporting conductive structures. This method offers a simple and controllable fabrication process with relatively high geometric precision [29,51]. However, the inherent high surface tension and poor wettability of LMs pose significant challenges during printing. To address these issues, Park et al. exploited the naturally formed oxide layer on the surface of LM, which plays a critical role in structural support, to develop a high-resolution LM printing technique (Figure 3a). This method allows for the direct printing of EGaIn into subcellular-scale (approximately 5 μm in diameter) soft neural probes and the fabrication of interconnect circuits on the surface of the skull, without requiring any annealing or post-processing. This feature provides a fundamental advantage over conventional metallic inks, which often require binders or post-processing to achieve similar functionality [46].

To overcome the challenges posed by the high surface tension and poor wettability of LMs, researchers have developed composite inks by dispersing LMs into micro and nanoscale particles. These inks take various forms, such as high internal phase emulsions, hydrogels, and thermoplastic encapsulations, effectively enhancing printing stability and controllability [30,47,52,53]. One strategy involves incorporating cellulose nanofibers and waterborne polyurethane into LM systems to form an interpenetrating polymer network, significantly improving ink flexibility and printing precision during DIW. The resulting composite conductors exhibited excellent electrical responsiveness after 500 cycles of strain, even with a high LM content of 78%, providing a fully automated and environmentally friendly route for complex circuit fabrication [53]. To meet the demand for instant conductivity in flexible electronics, a sintering-free, smear-resistant conductive composite ink was proposed. This ink embeds liquid metal microparticles (LMPs) and carbon black as conductive bridges within an entangled silicone elastomer matrix, achieving excellent electrical performance, smear resistance, and substrate adhesion. It enables direct printing on diverse surfaces such as PET, paper, and elastomers to form deformation-resistant, encapsulation-free conductive patterns—an essential technology for the rapid integration of soft electronic modules [54]. Another approach involved the chelation of calcium ions with polyacrylic acid and LM nanoparticles to construct a conductive hydrogel with self-healing and multifunctional sensing capabilities. This hydrogel supports precise recognition of electrocardiogram and electromyogram signals, making it suitable for wearable medical systems [55].

Currently, DIW-based 3D printing is progressively evolving from planar to three-dimensional structures, from rigid to flexible substrates, and from single-function components to multifunctional systems. Continuous innovation in structural materials, printable inks, fabrication strategies, and application scenarios is driving the development of wearable electronics, neural interfaces, and flexible energy systems toward greater precision, integration, and intelligence. As shown in Figure 3b, Lin et al. developed a high internal phase emulsion gel ink using a Carbopol hydrogel matrix, enabling 3D printing via DIW. The specific interaction between Carbopol and the surface oxide layer of the LM allows for stable dispersion of LM droplets at a high volume fraction (82.5%), resulting in a structurally robust emulsion gel. During the printing process, the hydrogel acts as a lubricant, reducing inter-droplet friction and preventing rupture of the oxide layer, thereby enhancing the printability of high-resolution, self-supporting LM structures. Additionally, the polyelectrolyte nature of Carbopol supports electrocapillarity, enabling rapid activation of electrical conductivity at low voltages and endowing the printed architectures with excellent electrical performance [47].

Lee et al. developed an omnidirectional 3D printing technique based on an LM-conductive filler composite system for constructing skin-conformal electronic devices. The formulated ink consists of a soluble solvent-emulsified Ag/MWCNT composite elastomer and an insoluble diethylene glycol phase, offering excellent printability and anti-clogging performance. This ink enables the printing of freestanding, filamentary, and out-of-plane structures with a minimum feature size below 100 μm and a stretchability exceeding 150%. The evaporation of the insoluble solvent promotes the aggregation of metal particles on the microporous surface, forming a highly conductive network with a conductivity as high as 6682 S cm^−1^ [56]. In addition, wearable RFID tags and miniature wireless light-emitting devices were fabricated using 3D direct-write microchannel technology, demonstrating the broad adaptability of LMs in flexible microfluidic circuits [57]. To enhance the integration of structural and functional properties, near-infrared-responsive LM nanoparticles were incorporated into resin systems to achieve 4D printing, thereby advancing the development of soft robotics and programmable materials [58]. A metal–phenolic network was introduced into a photothermal-responsive printing system to fabricate high-efficiency solar evaporators, offering a novel paradigm for integrated “water–energy–agriculture” systems [59]. Furthermore, a high-solid-content LM–CNF ink was developed for multilayer circuit board fabrication, with through-hole filling ensuring electrical continuity between conductive layers [60].

### 2.2. Embedded Printing of Liquid Metal

The high surface tension and low adhesion of LMs make it inherently difficult to form stable structures in free space, particularly self-supporting three-dimensional networks. To address this limitation, embedded 3D printing has emerged as a core strategy by depositing LM into rheologically tunable support media. This approach leverages hydrodynamic control, interfacial solidification, and material hybridization to enable the construction of complex flexible electronics, sensing devices, and soft robotics. Wu et al. proposed the use of a yield-stress support bath composed of acrylamide and nanoclay, enabling one-step deposition of LM wire structures (Figure 3c). Assisted by H_2_O_2_-induced oxidation, a surface oxide skin forms during printing, allowing high-fidelity, high-resolution (150 μm) 3D printing of LM networks. After UV curing, the resulting composite structure exhibited approximately 500% stretchability and excellent electrical stability, making it suitable for applications such as flexible resonators and three-dimensional electrodes [48]. Another strategy, termed electrochemical embedded 3D printing (3e-3DP), applied a voltage of 0.5–1.0 V within a hydrogel support matrix to induce the formation of a 100–300 nm gallium oxide layer. This enabled the fabrication of freestanding metal filaments with diameters of approximately 300 μm. After removing the supporting medium, the metallic conductors remained structurally stable and could be embedded into elastomers for the fabrication of flexible sensors and conductive components, exhibiting excellent robustness and adaptability [61]. Additionally, a liquid-phase 3D printing method was proposed for the rapid fabrication of conductive structures using LMs. In this method, a metal alloy with a melting point slightly above room temperature is employed as the printing ink and injected into a cooling liquid, significantly improving cooling rates and printing efficiency while preventing oxidation in air. The method successfully produced conductive patterns with diverse dimensions and complex geometries, and the effects of cooling fluid properties, injection pressure, and nozzle diameter on print quality were thoroughly analyzed [62].

A hybrid manufacturing approach combining 3D printing, vacuum casting, and encapsulation processes was proposed to fabricate LM/elastomer lattice materials. These materials utilize the phase transition behavior of LMs to achieve shape memory, stiffness modulation, and reconfigurable functionalities, showing promising applications in aerospace, tunable metamaterials, and robotics [63]. An LM–ceramic composite was developed by introducing EGaIn nanoparticles into a ceramic precursor and utilizing DLP printing to successfully construct tunable microwave-absorbing devices, offering a new route for integrating structural, electromagnetic, and printing functionalities [64]. In the development of tactile sensors, a two-step DLP printing process was employed to co-fabricate the substrate and microchannels, into which LM was injected to form sensing structures. A Wheatstone bridge circuit was used to decouple force and temperature signals, demonstrating excellent response performance in finger-grasping tests [65]. Further, a liquid metal silicon (LMS) ink with catalytic diffusion effects was introduced, enabling self-encapsulated embedded printing of triboelectric tactile sensors (Figure 3d). The resulting SEFTS device exhibited high sensitivity (0.308 V/kPa), strong linearity (R^2^ = 0.99), and remarkable stability (>10^4^ cycles), enabling integrated posture recognition and soft robotic perception [49]. To further improve ink stability and functional responsiveness, a Pickering emulsion strategy was used to fabricate carbon dot-armored LM nanodroplets with photothermal responsiveness and photoluminescence. These nanomaterials were employed in DLP printing to construct 3D architectures exhibiting stimulus-responsive and programmable deformation behaviors under laser or water stimuli, providing an innovative pathway for intelligent shape-morphing systems [66].

Embedded LM printing technology is evolving from two-dimensional filling to fully integrated three-dimensional architectures. Strategies now encompass oxide control, electrochemical modulation, self-encapsulation, and photoresponsive material systems. Supported by advances in support media design and multi-material printing platforms, embedded printing is poised to become a core technology in the manufacturing of flexible electronics and intelligent systems.

### 2.3. Extrusion and Infiltration Printing of Liquid Metal

The low viscosity of LMs allows them to easily infiltrate fine microchannels, while their exceptionally high electrical conductivity ensures outstanding electrical performance of the resulting microstructures, offering unique advantages in microchannel filling and microscale structure fabrication [67,68]. Compared to direct writing and embedded strategies, extrusion/infiltration-based LM 3D printing demonstrates superior material compatibility and process adaptability, making it particularly effective for fabricating multifunctional flexible devices with tightly coupled structural and functional properties. This approach employs methods such as mold injection, co-extrusion, and microstructure filling, leveraging the excellent flowability and wettability of LMs to integrate high electrical conductivity, thermal performance, and complex geometries into cohesive architectures [42,45]. Yu et al. proposed an LM skeleton elastomer based on a dual-continuous gyroidal microstructure (Figure 3e). By employing a fused deposition modeling (FDM) printed sacrificial mold combined with LM injection, they fabricated a deformable thermal regulator that simultaneously exhibits high thermal conductivity and excellent stretchability [50]. Building on this foundation, as shown in Figure 3f, the team further introduced a volumetric metallization strategy for 3D-printed composites. By precisely controlling the pore architecture and applying low-pressure vacuum assistance, they achieved complete infiltration of gallium-based LM into FDM-printed acrylonitrile–butadiene–styrene (ABS) scaffolds. The resulting structure features dual-continuous phases and anisotropic coupling of mechanical, thermal, and electrical properties. Experimental measurements revealed a thermal conductivity of up to 25.3 W/m·K, tensile strength of 35.4 MPa, and electrical conductivity exceeding 10^6^ S/m, demonstrating broad applicability in microelectronics, radio-frequency devices, and thermal management systems [44].

Ames et al. systematically investigated the fabrication mechanism of extruded LM core–shell structures. By controlling the core-to-shell area ratio to 0.37, they significantly enhanced structural stability and electrical uniformity, enabling reliable printing of curved and cantilevered paths suitable for flexible cables and robotic actuators [69]. Lu et al. proposed an LM emulsion gel strategy, in which densely packed microcapsules were synthesized via a two-step method and dispersed within an elastic matrix to form an extrusion ink with a strain range up to 1000% and electrical conductivity of 2.2 × 10^4^ S/cm. This ink was successfully applied in 3D printing of NFC tags and LED displays [70]. Zhang et al. employed coaxial printing to fabricate LM-core/magnetically responsive shell structures that combine high electrical conductivity (2.07 × 10^6^ S/m) with favorable mechanical modulus (0.87 MPa), demonstrating strong potential for integrated structural–functional applications in magnetic actuators and minimally invasive catheters [31]. Shan et al. introduced a universal medium printing framework, categorizing the physicochemical interactions between LMs and various surrounding media, thereby expanding environmental adaptability [71]. Feig et al. proposed a brittleness-induced strategy to achieve controlled degradation for implantable devices [72]. Li et al. demonstrated the functional expansion of LM–hybrid hydrogel scaffolds in antibacterial activity and tissue healing [73]. Furthermore, ongoing advancements in LM applications within flexible integrated circuits and wearable electronics continue to gain momentum. Current research focuses on pattern design [74], electrode fabrication [75], functional inks, and multi-material printing strategies, pushing the field toward higher resolution and multifunctional integration.

Liquid metal printing technology, as an emerging additive manufacturing method, is showing disruptive potential in the fields of fluorescent display and flexible electronics. Its core advantage lies in the precise control of the deposition path [76] and surface functionalization modification of LMs [77], which can simultaneously achieve device integration of micro scale structure construction and multi physical field coupling, providing a breakthrough solution for the development of new optoelectronic systems [78].

In summary, different LM 3D printing strategies exhibit distinct advantages and limitations, as outlined in Table 2. Direct-ink writing offers high precision and facile control, but its stability is hindered by oxidation, making it more suitable for microscale applications such as neural probes and interconnects [46]. Embedded printing ensures high stretchability and structural fidelity within a supporting matrix, yet the relatively slow printing speed and low throughput restrict its scalability, while its strength lies in bioelectronics and morphing systems [61]. Extrusion and infiltration printing demonstrate excellent material compatibility and rapid fabrication, though the trade-offs include lower resolution and limited reusability of the substrate, making them more appropriate for thermal management devices and microelectronic applications [50]. Overall, these complementary techniques highlight the necessity of balancing resolution, stability, speed, and application-specific demands in advancing LM-based additive manufacturing.

## 3. Liquid Metal Catalysis

In the field of catalytic reactions, traditional solid-state metal catalysts often face challenges such as insufficient catalytic activity and limited selectivity in complex reaction environments due to their fixed active site positions and rigid structural properties. This atomic-level static configuration restricts the flexible adsorption of reactants on the catalytic surface and the modulation of reaction pathways, making it difficult to adapt to variable reaction conditions [32]. Additionally, solid-state metal catalysts commonly encounter issues such as complex preparation processes, limited structural precision, and deactivation caused by the coverage of active sites by by-products during preparation, further constraining their application performance and service life. Therefore, the development of novel catalytic material systems with dynamic tunability, high stability, and high adaptability has become a key research focus [79,80].

LM catalysis has garnered widespread attention and rapid development in recent years due to its high fluidity, highly active interfacial atoms, and excellent electron transport properties [27]. The interfacial atomic arrangement of LMs exhibits a dynamic disordered state, which differs from the static structure of solid metals. This characteristic endows LMs with multiple advantages in catalytic reactions. Firstly, the interface composed of high-entropy liquid atoms contains abundant active sites, significantly enhancing catalytic reaction rates and interfacial reactivity [81]. Secondly, the atomic structure of LMs possesses self-adaptability, enabling real-time adjustments in response to changes in temperature, electric fields, or chemical environments during the reaction process, which facilitates improved catalytic selectivity and pathway control. Based on these considerations, this paper categorizes the applications of LM catalysis into two typical mechanisms: direct catalytic reactions and indirect catalytic reactions based on LM skeletons. It systematically reviews the research strategies, structural designs, mechanistic explorations, and representative achievements in each category, aiming to provide a comprehensive reference and insights for future research directions in this field.

### 3.1. Liquid Metal Direct Catalytic Reaction

We define the reactions where LM or its alloys are directly employed as catalysts, or where active sites are formed through alloying to dominate the catalytic reaction process, as direct catalysis. The essence of direct catalysis by LM stems from its unique physical state and chemical properties. Firstly, the atoms in LM are unconstrained by lattice structures and exhibit highly dynamic arrangements. This degree of freedom endows its surface with exceptional adaptive capacity [82]. During catalysis, the LM surface can respond in real-time to the adsorption requirements of reactant molecules (e.g., gases, organic compounds) by undergoing atomic-scale structural oscillations and orientation adjustments, enabling dynamic reconstruction of active sites. This process reduces reaction energy barriers and enhances the conversion efficiency of intermediates. Secondly, the high electrical conductivity and electron migration characteristics of LM are crucial. Its internal free electrons can rapidly respond to charge transfer demands driven by electrochemical or thermochemical processes, forming localized electric fields or promoting electron-hole separation, thereby directly driving redox reactions [83]. Additionally, the synergistic interaction between LM and doped metals (e.g., Cu, In, Sn) can reconstruct active centers, further optimizing the selectivity and rate of reaction pathways through intermetallic charge transfer or alloying effects.

**Figure 4:**
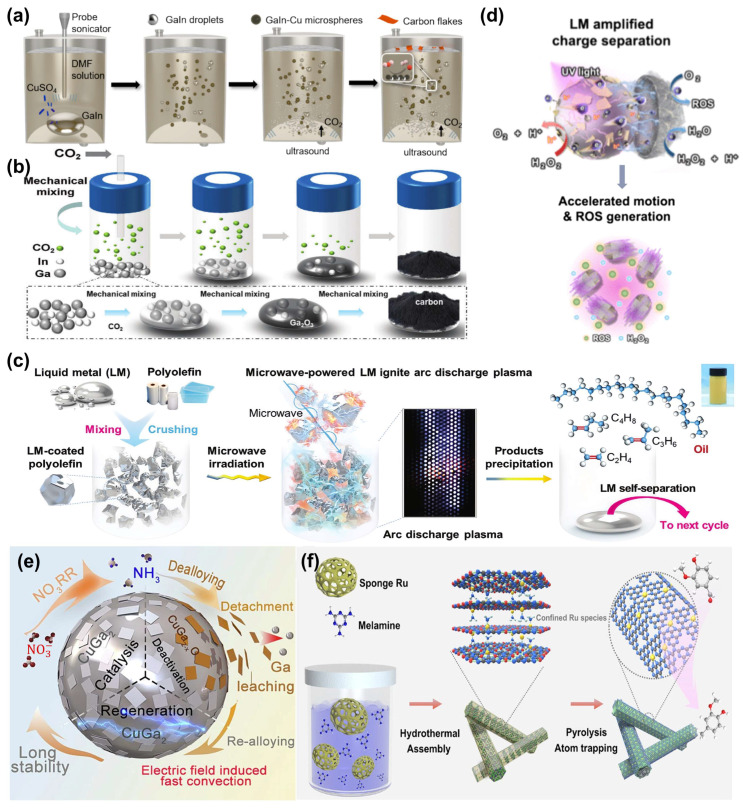
Illustrative diagram of the representative LM catalysis. (**a**): Fabrication of GaIn-Cu composite catalysts through ultrasonic-assisted methods, and CO_2_ conversion facilitated via prepared GaIn-Cu composite catalyst [87]. (**b**): The conversion of carbon dioxide into solid carbon using LMs [35]. (**c**): The working principle of microwave-powered LM depolymerization for polyolefin plastic upcycling [88]; (**d**): The LM-based photocatalytic MNMs for antibacterial therapy [89]; (**e**): The CuGa2-LM electrode for the electro chemical nitrate reduction [90]; (**f**): The synthesis and characterization of hollow Ru1@m-tube [91].

Under thermal energy driving, LM can catalyze the reduction of CO_2_ to produce carbon and oxygen (CO_2_ → C + O_2_) [84,85]. Based on this, Yu et al. designed a carbon dioxide capture and conversion platform using a helical coaxial countercurrent microreactor, employing DMF absorbent for cyclic CO_2_ adsorption and desorption, which was coupled with the LM catalytic process. The helical structure enhances mass and heat transfer by inducing secondary flows, significantly improving CO_2_ decomposition efficiency and carbon capture rate through enhanced gas–liquid interactions. The highly conductive GaIn-DMF interface accelerates electron transfer, promoting the aggregation of CO and O intermediates and facilitating their efficient catalytic reduction. This technology can utilize waste heat and solar energy to convert CO_2_ into high-value solid carbon, achieving synergistic benefits in carbon reduction and resource utilization [86]. Furthermore, Yu et al. developed a Cu-based catalytic system based on GaIn-Cu LM, which efficiently reduces CO_2_ to solid carbon at room temperature and atmospheric pressure (Figure 4a), with a conversion efficiency as high as 96%. This system exhibits low energy consumption, environmental friendliness, resistance to coking, and good recyclability [87]. Additionally, addressing the challenge of high energy consumption in CO_2_ conversion, Luo et al. designed a power-independent CO_2_ catalytic strategy that utilizes the triboelectric potential generated at the interface between LM and its oxide layer to achieve selective conversion of CO_2_ into carbon monoxide and carbon without the need for an external voltage (Figure 4b). This process highlights the mechanisms of spontaneous polarization on the LM surface and electron migration induced by localized electric fields. By leveraging the responsiveness of low-melting-point LMs to mechanical energy, this strategy can be driven by green energy sources such as wind and wave energy, significantly reducing energy consumption [35].

Parker et al. successfully prepared Cu–Ga droplets by ultrasonically dispersing LM into molten sodium acetate at high temperatures. The Cu–Ga droplets exhibited excellent performance in ethanol electrooxidation, demonstrating their potential in catalytic applications [92]. Murguía-Ceja et al. synthesized a Cu-doped In_2_O_3_–Ga_2_O_3_ heterojunction photocatalyst based on an In–Ga LM alloy using a precipitation-assisted hydrothermal method and systematically investigated the effect of Cu doping content on its performance. The catalyst showed significantly enhanced interfacial electronic state regulation ability and successfully achieved visible-light degradation of acetaminophen [93].

As shown in Figure 4c, Gao et al. proposed the use of non-equilibrium electromagnetic microwaves to activate EGaIn for the efficient depolymerization catalysis of polyolefin waste. EGaIn provides a highly active reaction interface and a self-renewing liquid surface, significantly improving the conversion efficiency of waste plastics such as polyethylene and polypropylene into high-value olefin monomers and hydrocarbon oils. Owing to its excellent chemical bond activation and anti-sintering properties, the system maintained an oil yield above 81 wt.% over 30 cycles, with a turnover frequency as high as 2.83 kg_Plastic_ mL^−1^ [88]. Zhai et al. found that LM can act as a self-heater, mechanical agitator, and catalyst under an alternating electromagnetic field, enabling efficient thermal depolymerization of polyesters. By alloying Ga with metals such as Sn, Zn, Al, and Mg, they tuned the surface oxide layer and catalytic activity. Electromagnetic induction rapidly heated LM to 600 °C within 3 min and induced disturbances to enhance mass transfer. Without external heating or stirring, LM selectively depolymerized polycaprolactone into ε-caprolactone (>96% selectivity) at moderate temperatures, with a rate of 500 mg h^−1^ mL^−1^, and applied to various polyesters, demonstrating its catalytic efficiency and broad applicability [94]. Polo-Garzon et al. utilized low-melting-point LM (Ga, In–Ga) as catalysts for the controlled dechlorination of polyvinyl chloride (PVC) at 200 °C, achieving a 90% dechlorination rate. This approach generated H_2_ via close liquid–solid contact (accounting for ~11% of the hydrogen in PVC), avoiding the release of hazardous HCl gas. Chlorine was sequestered in the carbonaceous product and could be eluted with acetone. The products were easily separable, and the LM catalysts could be recovered and reused by cooling [95].

Leveraging the dynamic properties of LMs (surface self-adaptability, atomic migration) and intermetallic synergistic effects facilitates the stabilization of reaction temperatures and continuous regeneration of oxidation-poisoned catalytic surfaces. Bao et al. utilized molten Ga as a liquid catalyst for the decomposition of nitric oxide (NO) [96]. Crawford et al. found that Galinstan could serve as a highly efficient and stable electrocatalyst for the electrochemical conversion of nitrate to ammonia, achieving a Faradaic efficiency of 100%. The alloy is enriched to form active sites during the reaction, significantly suppressing the hydrogen evolution reaction and enhancing NH_3_ selectivity [97]. Karma et al. reported a Ga-based LM catalyst containing 2 wt.% Cu, which efficiently catalyzed the synthesis of ammonia from N_2_ and H_2_ under 4 bar pressure. Through the synergistic interaction between Cu and Ga, the LM catalyst exhibited superior catalytic performance compared to single metals. In situ spectroscopy and molecular dynamics simulations revealed that the migration behavior of Cu atoms in the liquid environment significantly promoted the dissociation of H_2_ molecules, providing new insights into the applications of LMs in important reactions such as ammonia synthesis [98].

In summary, LM-based direct catalytic reactions leverage the dynamic and self-adaptive liquid surface, high electrical conductivity, and synergistic alloying effects to overcome the intrinsic rigidity and deactivation issues of traditional solid catalysts. These properties enable efficient CO_2_ reduction, selective polymer depolymerization, pollutant degradation, and ammonia synthesis, with remarkable activity, selectivity, and recyclability. The capacity of LMs to sustain long-term catalytic stability through self-regeneration of active sites highlights their promise as next-generation catalysts for energy conversion, environmental remediation, and green chemical production. These advances not only demonstrate the practical feasibility of LM direct catalysis but also provide critical mechanistic insights that guide the design of dynamic catalytic systems [32,99,100].

### 3.2. Liquid Metal Indirect Catalytic Reaction

When employed as a support or matrix, LMs exert catalytic effects primarily through interfacial interactions and electronic structure modulation. On one hand, the high surface entropy and fluidity of LMs can stabilize loaded active metals (e.g., Pt, Ru, Cu), preventing their aggregation or deactivation. The liquid matrix anchors the active metal atoms onto its surface via van der Waals forces or chemical bonding, forming atomically dispersed catalytic sites. Simultaneously, the inherent fluidity of the LM enables dynamic renewal of active sites, avoiding catalyst poisoning caused by excessive adsorption of intermediates or products [101]. On the other hand, interfacial charge transfer between the LM and the active metal is a key mechanism. The liquid matrix modifies the electronic density of the states of the active metal via electron spillover or d-band tuning, thereby optimizing the adsorption strength of reactants (e.g., H_2_, CO_2_) and lowering the activation energy for intermediate transformation (e.g., CO, CHO). Additionally, the dielectric properties and surface polarity of LMs can modulate the reaction microenvironment—for example, by tuning solvation effects or acid-base characteristics—which further influence the catalytic activity and selectivity [80].

As shown in Figure 4d, Guo et al. proposed a facile method for preparing an LM photocatalytic micro/nanomotor by compounding LM with graphitic carbon nitride and coating it with a Pt layer, which significantly enhanced the charge separation efficiency (by over 6.5-fold) and migration efficiency under illumination. This structure exhibited self-electrophoretic propulsion capability in a H_2_O_2_ solution, with light illumination further enhancing its motility and reactive oxygen species generation performance [89]. Moritz et al. systematically explored a ternary LM catalyst system (SCALMS) with gallium as the matrix and platinum as the active site, while introducing tin or indium to modulate its catalytic performance. The study revealed that SCALMS demonstrated excellent activity and stability in high-temperature reactions such as alkane dehydrogenation, attributed to its liquid fluidity and atomically dispersed catalytic sites. The introduction of a third metal significantly adjusted the surface composition, thereby altering the catalytic reactivity [102].

The fluidity of LM facilitates sufficient contact between supported active sites and reactants while suppressing product aggregation. Long et al. anchored Pt atoms in a Ga matrix for fatty acid hydrogenation, achieving 100% conversion and 98.7% selectivity toward octadecane [103]. Cao et al. developed a flowable CuGa_2_-loaded LM (CuGa-LM) catalyst for the electrocatalytic reduction of nitrate to ammonia (Figure 4e). The catalyst leveraged the excellent surface fluidity of LM to enhance mass transfer efficiency and significantly improve catalytic performance. Catalyst deactivation caused by CuGa_2_ segregation during the reaction was resolved via a rapid interfacial realloying strategy, resulting in a 1500% increase in catalytic lifespan [90]. Rahim et al. proposed using room-temperature liquid Ga as a matrix to dissolve Pt, constructing a low-temperature liquid Pt catalytic system that overcomes the limitation of Pt requiring high temperatures to liquefy. In this system, Pt atoms are dispersed in Ga without forming Pt–Pt bonds and can effectively drive various catalytic reactions. In a Ga–Pt system containing only 0.0001 at% Pt, the mass activity for methanol oxidation exceeded that of commercial Pt/C catalysts by three orders of magnitude. Furthermore, the isotropic and dynamic behavior of Pt atoms in Ga endowed the system with excellent anti-deactivation properties [104].

Song et al. developed a single-atom catalyst based on an LM catalytic strategy, as illustrated in Figure 4f. This catalyst was prepared by coating GaInSn onto the surface of hollow carbon nitride loaded with ruthenium (Ru). The LM can donate electrons to the sparsely distributed Ru sites, significantly modulating the catalytic pathway and achieving a reversal in the selectivity of vanillin hydrogenation. Within 5 h, the catalyst achieved nearly 100% conversion of vanillin and a remarkable 93.8% selectivity toward the target product, vanillyl alcohol [91]. Zhou et al. found that introducing low-melting-point LM gallium into ZSM-5 zeolite significantly enhanced its catalytic performance in methanol-to-hydrocarbons reactions. The liquid gallium effectively prolonged the catalyst lifetime by slowing down coke deposition and promoting the desorption of carbonaceous species, extending its operational duration to approximately 14 times that of conventional ZSM-5 [105].

Using acylation reactions over zeolite catalysts as a model, Zhou et al. discovered that LM could significantly enhance the selectivity of esterification products while suppressing side reactions. The strong interaction between LM and zeolite in this system could modulate acidic sites, effectively overcoming challenges such as low yields, excessive by-products, and catalyst deactivation typically encountered in traditional solid acid catalysis [106]. Zhang et al. developed a structurally tunable Cu-doped LM catalyst (Cu-LMC), which exhibited high yield and selectivity (82%) in the conversion of methane to methanol. The liquid Ga matrix endowed the catalyst with fluidity and surface adaptability, facilitating the formation of a Cu-O-Ga configuration. This catalyst effectively optimized the reaction pathway by reducing the desorption energy of products and inhibiting side reactions [33].

LM catalysis, as an emerging and cutting-edge field, holds a core advantage in breaking through the limitations of activity and stability inherent in traditional solid-state catalysts. Direct catalytic systems leverage the dynamic atomic arrangement, high electrical conductivity, and intermetallic synergistic effects of LMs to achieve efficient regulation of complex reaction pathways, such as CO_2_ reduction and ammonia synthesis. Indirect catalytic systems, on the other hand, significantly enhance the activity and selectivity of reactions like single-atom catalysis and photocatalysis by utilizing the liquid matrix to modulate charge transfer to active metals, stabilize interfaces, and optimize microenvironments. Together, these two approaches address common pain points of traditional catalysts, such as susceptibility to aggregation, poisoning, and poor adaptability, demonstrating unique advantages in electrocatalysis, thermal catalysis, and material synthesis. 

## 4. Liquid Metal-Based Sensor

The unique physical properties of LMs—including high electrical and thermal conductivity, biocompatibility, and deformability—grant them significant advantages in the fields of flexible electronics and wearable sensing. Compared to traditional rigid sensors, LM-based sensing devices exhibit intrinsic flexibility, stretchability, and strong environmental adaptability, enabling effective operation in complex scenarios such as conformal contact with curved surfaces and dynamic deformation monitoring. These characteristics make LM sensors particularly valuable for applications in health monitoring, human–machine interaction, and soft robotics. This section focuses on recent research advances in the applications of functional LM materials in sensing technologies, providing a systematic review from three perspectives: mechanical sensing, chemical sensing, and multimodal sensing. By summarizing and analyzing relevant studies, this review aims to provide a comprehensive understanding of the current state and emerging trends of LM-based sensors, offering insights and guidance for future research and practical applications.

### 4.1. Liquid Metal-Based Mechanical Sensor

Mechanical response sensors detect external forces (such as pressure, deformation, or temperature variations) through regular changes in electrical parameters. Such devices must meet core requirements, including a flexible substrate, tolerance to large deformations, long-term stability, and precise sensing capabilities. LMs, owing to their unique physical properties, serve as ideal material foundations for realizing such devices: their inherent fluidity enables the sensor structures to dynamically reconfigure in real time in response to external forces, while their high intrinsic conductivity ensures a strong correlation between mechanical stimuli and electrical signals. Notably, LM-based systems exhibit topological reconfigurability and self-healing capabilities, providing material assurance for the development of long-lifetime flexible sensors suitable for continuous monitoring scenarios.

#### 4.1.1. Liquid Metal-Based Strain Sensor

**Figure 5:**
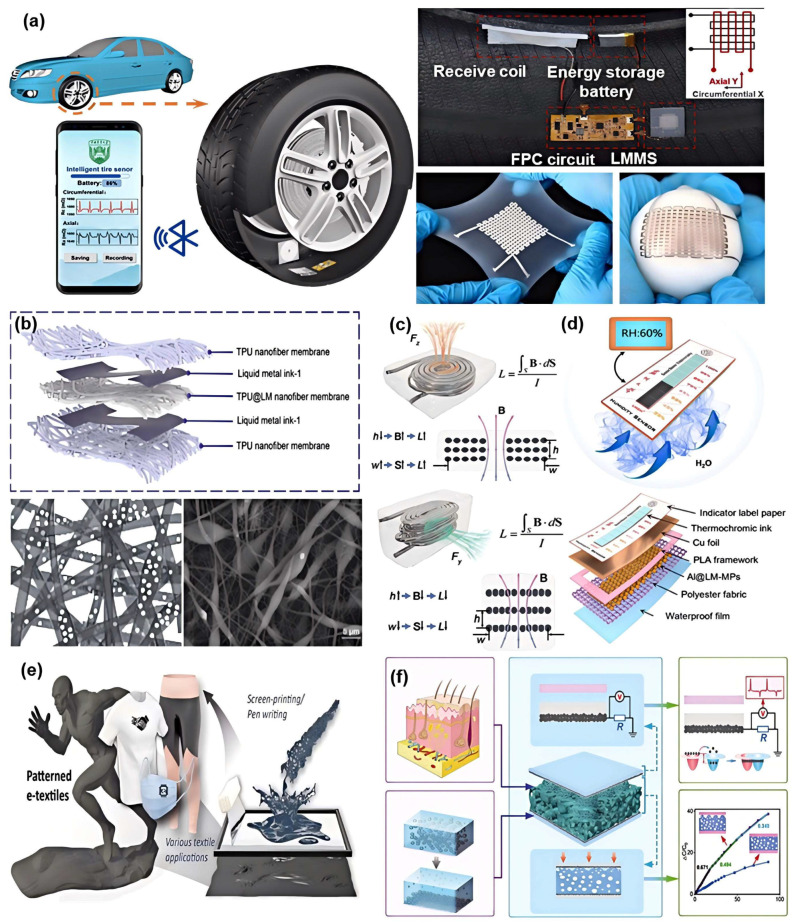
Schematic representation of typical LM-based sensors. Mechanical sensor: (**a**): The working mechanism and structure of LMSCS for smart tire strain monitoring with its applications [108]; (**b**): TPU@LM sensor with its fiber dielectric layer and SEM image [110]; (**c**): Anisotropic sensing capabilities of AI-LMS [111]; Chemical sensor: (**d**): LM microparticle enabled humidity sensor [112]; Multimodal sensor: (**e**): Synthetic roadmap to convert bulk LM to SeLM-SeCNT nanodroplets and the fabrication of water-based SeLM-SeCNT conductive [113]; (**f**): The multifunctional flexible sensor [114].

As core sensory components for capturing deformation and motion states, strain sensors hold irreplaceable technological value in cutting-edge fields such as health monitoring, human–machine interaction, and soft robotics. Their primary function is to convert mechanical deformation into quantifiable electrical signals. Owing to their distinctive liquid-phase characteristics and high electrical conductivity, LMs have emerged as ideal materials for fabricating high-precision, stretchable strain sensors. By integrating microfluidic channels, composite encapsulation, and multilayer circuit designs, the structure of LM-based strain sensors can be flexibly tailored to meet specific application requirements [107]. Dong et al. proposed a flexible liquid-metal-based solution that integrates sensing and energy functionalities (Figure 5a), marking the first application of microfluidic technology in smart tire strain monitoring [108]. The developed LM sensor and coil exhibited remarkable stretchability (over 400% tensile strain) and durability (over 5000 stretching cycles), effectively addressing the mechanical mismatch between rigid electronic interfaces and the tire’s complex inner surface during prolonged use. The sensor consists of multilayer LM sensing networks, enabling simultaneous circumferential and axial micro-strain monitoring with high linearity (R^2^ = 0.99744) and a strain resolution of 30 μm. A custom wireless power module, based on magnetic resonance coupling, provides continuous energy to the sensor. Combined with Bluetooth 5.0, real-time sensor data are transmitted to a dedicated mobile application, enabling dynamic visualization of tread deformation waveforms. Inspired by the principle of water valve regulation, Yao et al. developed a highly sensitive, wide-range, and fast-response stretchable strain sensor [109]. By embedding rigid PDMS convex structures within a soft elastomer substrate, the deformation behavior of the LM-filled conductive channels is effectively modulated. Under strain, the soft matrix undergoes significant deformation while the rigid convex structures remain relatively unchanged, leading to a pronounced contraction of the conductive channel. This design strategy enhances sensing sensitivity by two orders of magnitude.

In the fabrication of LM-based sensors, beyond microfluidic strategies, dispersing LMs into micro- and nanoscale particles with surface functionalization or forming composites with other functional materials has become a crucial pathway to enhance overall sensor performance. These approaches utilize microstructural design to effectively resolve the trade-off between conductivity and stretchability under high mechanical deformation, thereby expanding the application boundaries of LMs in the field of flexible electronics.

In terms of conductivity-enhanced composite sensors, the LM/CNT/PDMS ternary system developed by Kim’s team achieved a three-dimensional conductive network through the incorporation of 1.5 wt.% CNTs, resulting in a gauge factor of 5.35 within the 50–100% strain range while also imparting electromagnetic wave absorption properties [115]. The sensor demonstrated a low hysteresis error (<5%) under cyclic loading at 80% strain, validating its reliability for continuous monitoring in wearable medical devices. Magnetic particle-based composite strategies have further opened new avenues for skin-like flexible sensing. Zhang’s team dispersed 40 μm nickel particles into an LM matrix and induced alignment via an external magnetic field to establish a resistance modulation mechanism [116]. The sensor, doped with 12 wt.% nickel particles, exhibited a gauge factor of 5.17 at 300% strain, and maintained a conductivity degradation rate below 8% after 2000 stretching cycles. A 14-channel array design, combined with a quadratic discriminant analysis algorithm, enabled 100% recognition accuracy of sign language gestures, offering a low-cost solution for flexible human–machine interaction interfaces. Wang’s team designed an SA-modified LM hydrogel that maintains 382% stretchability and a gauge factor (GF) of 3.66 even at −40 °C, broadening its applicability in low-temperature wearable electronics [117]. Additionally, a fiber-optic sensor composed of an LM/CNF coating combined with a PUT substrate demonstrated a rapid response time of 304 ms and a GF of 73.7 ± 3.9, making it suitable for high-precision human physiological monitoring [118]. For clarity of comparison, Table 3 summarizes representative LM-based strain sensors, emphasizing their stretchability, gauge factor, and stability. This comparative overview enables a direct assessment of material–property relationships and highlights the suitability of different sensor architectures for targeted application scenarios.

#### 4.1.2. Liquid Metal-Based Pressure Sensor

Pressure sensing is one of the most significant application areas for functional LM materials. Through innovative structural designs and composite material strategies, researchers have developed a series of high-performance LM pressure sensors, achieving continuous advancements in sensitivity, detection range, and stability. Capacitive pressure sensors have attracted considerable attention owing to their advantages of simple structure, low power consumption, and high sensitivity. As shown in Figure 5b. Xu et al. developed a fully fibrous anti-interference capacitive pressure sensor for human health monitoring. This sensor employs a hybrid electrospinning technique to fabricate a fiber-based dielectric layer. By compounding modified photonic fiber particles with a TPU elastomer, the electrical properties of the dielectric layer are significantly enhanced, and excellent anti-interference capability is imparted. Its innovative fully fibrous structural design effectively reduces the Young’s modulus of the functional layer, enhances its tensile deformation capacity, and achieves a detection range of up to 2100 kPa. Testing indicates that, under electromagnetic interference, its signal-to-noise ratio (SNR) improves nearly threefold compared to sensors with a pure TPU dielectric layer, and it maintains stable repeatability after 20,000 loading-unloading cycles [110].

Inspired by human fingertips, Li et al. developed an anisotropic inductive LM sensor (AI-LMS) using Ecoflex or PDMS substrates combined with EGaIn LM to construct flexible coils for multi-axis pressure detection (Figure 5c) [111]. The AI-LMS demonstrated opposite inductive responses to normal and lateral pressures and outperformed resistive signals in terms of linearity, resolution, and stability, with a response time of only 0.15–0.4 s. When integrated with deep neural networks, the sensor achieved effective signal decoupling and significantly expanded the detection range, successfully enabling high-precision 3D scanning and real-time recognition of robotic grasping behaviors. Lee et al. proposed a tunable pressure sensor based on gallium–elastomer composites, utilizing the solid–liquid phase transition of gallium microparticles to achieve dual-mode sensing. In soft mode, the sensor exhibited a sensitivity of 16.97 kPa^−1^, while in rigid mode, the bandwidth extended up to 1.45 MPa, covering a broad detection range from 3 Pa to 1.45 MPa [119]. The sensor demonstrated high inter-device consistency, with a minimum detectable pressure 97% lower and a maximum detectable pressure 262.5% higher than that of human skin (100 Pa–400 kPa), breaking the conventional trade-off between sensitivity and bandwidth. When integrated into a 2D array, it formed adaptive robotic skin capable of real-time pressure sensing and diverse applications.

LMs have demonstrated multiple advantages in the field of strain sensing. Yang et al. developed a flexible structure by embedding LMs into foam walls, achieving excellent compressibility and dielectric tunability. Luo et al. coated gallium–indium alloy onto a triply periodic minimal surface (TPMS) lattice to construct a three-dimensional conductive network. This structure generated resistance changes under mechanical deformation and exhibited outstanding electromechanical responsiveness, fast response time, and long cycle life [120]. Li et al. proposed a flexible sensing array composed of LM microelectrodes and sensing units, achieving high sensitivity (7.42 kPa^−1^), a low detection limit (0.84 Pa), and rapid response (54 ms). The device accurately mapped surface pressure and achieved a recognition accuracy of 99.7% using machine learning algorithms [121]. Gong et al. developed an LM–graphene composite conductive structure that significantly enhanced sensitivity to subtle deformations, enabling monitoring of various physiological signals [122]. Wang et al. fabricated lightweight, flexible electrodes through sedimentation and solidification of LMs and combined them with carbon nanofiber foam to form a strain sensor with a sensitivity of 3.8 kPa^−1^ and a response time of 83 ms [123]. Wu et al. constructed a flexible magnetoelectric sensor that generated an induced voltage under deformation (up to 45.6 µV) and exhibited a sensitivity of 10.9 kPa^−1^ [124]. Kouediatouka et al. developed a CNT/LM-coated thin-film sensor that showed high gauge factors (20.6–57) and fast response times (70 ms), making it suitable for high-precision wearable strain sensing applications [125]. Representative performance metrics of these LM-based pressure sensors are summarized in Table 4, highlighting their versatility in pressure mapping, physiological monitoring, and wide-range detection.

### 4.2. Liquid Metal-Based Chemical Sensor

With the rapid advancement of technology, the demand for chemical sensors is increasing across various fields such as environmental monitoring, healthcare, and industrial manufacturing. However, conventional chemical sensors are increasingly limited in terms of performance, flexibility, and adaptability, making it difficult to meet the needs of complex and dynamic application scenarios. As emerging materials, LMs offer unique physicochemical properties that present new opportunities and breakthroughs in the design and development of chemical sensors, gradually becoming a research hotspot in this domain. Chen et al. proposed a passive humidity sensor based on LM microparticles (Al@LM-MPs), achieving high sensitivity, low cost, and an ultra-compact form factor for humidity detection (Figure 5d). The core sensing mechanism involves the exothermic reaction between Al@LM-MPs and water molecules, which rapidly triggers a color change in thermochromic ink, thereby enabling visual display of humidity levels. The sensor operates over a range of 30–90% relative humidity (RH) with a response time of less than 4 s per 10% RH—approximately 41,700 times faster than traditional humidity cards—offering exceptional economic value and portability [112].

In wearable chemical sensing, Chen et al. developed a flexible electrochemical sensing system using unmodified GaInSn LM patterned on double-layer textiles. Polymethyl methacrylate (PMA) was employed to enhance adhesion between the metal and fabric, enabling direct patterning without surface pretreatment or complex equipment. Utilizing commercial electrodes for constant-voltage electrochemical detection, the system successfully detected glucose at the millimolar level in artificial sweat [126]. Further exploring multifunctional sensing systems, Wang et al. designed a biomimetic LM-based mechanoelectric device that mimics piezoelectric protein functions. By spatially configuring LM pillars and electrodes, the device converts mechanical input into electrical signals. Relying on the electrochemical properties of LMs, they feature a fully flexible architecture capable of generating reversible charge gradients and self-powered switching behavior [127]. In the domain of nanomaterials, Liu et al. developed a composite nanomaterial based on EGaIn, functionalized with p-phenylenediamine to inhibit oxidation and decorated with gold nanoparticles to enhance electrochemical performance. The material exhibited high electrical and thermal conductivity along with excellent photothermal properties, making it well-suited for wearable chemical sensors. A skin-mounted patch constructed from this material was capable of detecting C-reactive protein while simultaneously providing mechanical sensing and anti-inflammatory healing functions, highlighting its multifunctional potential in biochemical detection and therapy [128].

Nguyen fabricated a highly sensitive, low-temperature ammonia sensor by using an LM exfoliation method to synthesize 2D SnO_2_ nanosheets. These nanosheets, characterized by high specific surface area and superior electronic structure, enabled rapid NH_3_ detection across a concentration range of 5–500 ppm [129]. To address the need for volatile organic compound (VOC) detection in both gaseous and liquid environments, Kim proposed a fully flexible chemical sensing platform based on EGaIn, integrating a 3D soft inductive–capacitive sensor with a readout coil to enable battery-free, wireless VOC detection in both phases [130]. This platform utilizes EGaIn patterning to fabricate interdigitated capacitors and spiral inductors. Through microfluidic modulation of capacitance in response to dielectric changes in liquids, the system demonstrated effective VOC discrimination in the liquid phase. For gas-phase detection, the platform showed high sensitivity to model compounds such as isopropanol, ethanol, and methanol, outperforming conventional PDMS-membrane-based silicon sensors.

The applications of LMs in chemical sensor design are expanding rapidly, offering notable advantages in interfacial material tuning, 3D printed patterning, micro/nanostructure fabrication, and multimodal functional integration. With continued advancements in material systems and manufacturing techniques, LMs are poised to enable the development of more efficient, sustainable, and intelligent chemical sensing platforms across diverse fields such as smart wearables, biomedical diagnostics, and environmental monitoring.

### 4.3. Liquid Metal-Based Multimodal Sensor

With the rapid development of flexible electronics and wearable devices, new requirements have been put forward for sensors to be self-powered, sustainable, and multifunctional integrated [131]. LM has become an ideal material for constructing self-powered sensing systems due to its excellent conductivity, fluidity, and biocompatibility [132]. This type of sensor can not only achieve high sensitivity perception of multimodal signals such as pressure, strain, and temperature, but also convert mechanical and thermal energy in the environment into electrical energy through mechanisms such as triboelectric nanogenerator (TENG) [113] and thermoelectric conversion [133], providing continuous power for itself and eliminating dependence on external power sources.

In the field of energy harvesting and self-powered sensing, multiple studies have demonstrated the remarkable advantages of LM. Chung et al. developed a water-based conductive ink composed of LM, CNTs, and natural biopolymers for textile-structured electronic devices (Figure 5e). This device not only achieved a wide strain response range (gauge factor up to 46.04) and temperature sensing capability, but also integrated a TENG unit, which harvested energy from human motion and delivered a power density of 162 mW/m^2^. Such integration enabled pressure sensing without the need for external power, thereby providing continuous energy for health monitoring [113]. Zhu et al. proposed a multifunctional sensor with a sandwich-like structure, in which the upper and lower layers consisted of PDMS/LM composite electrodes and the middle layer was a porous PDMS/CNT dielectric. In this design, the LM electrodes simultaneously functioned as sensing units and TENG components, enabling not only highly sensitive pressure detection but also direct conversion of mechanical stimuli into electrical signals, thus achieving self-powered dynamic force sensing and material recognition [114]. Luo et al. stabilized LM using Tween 80-modified multi-walled carbon nanotubes and hyaluronic acid, constructing a polymerizable LM composite to fabricate a conductive hydrogel. The hydrogel exhibited excellent stretchability (1192%), adhesiveness, and conductivity (0.78 S/m), enabling its use as a strain sensor for real-time joint motion monitoring. Additionally, it served as a flexible triboelectric nanogenerator electrode, integrated with a microcontroller for self-powered operation and wireless transmission of motion data [134].

In addition to TENG, LM has also been used to construct thermoelectric self-powered systems. Cui et al. developed a porous LM/carboxymethyl chitosan (CMCS) hydrogel that not only has excellent sensing performance (response time of approximately 22 ms), but also utilizes the photothermal effect of LM to achieve thermoelectric conversion, directly converting heat difference into electrical energy, making it suitable for energy-autonomous sensing systems in wearable scenarios [133].

In addition, many studies have also been devoted to improving the environmental adaptability and functional integration of LM-based sensors. Zou et al. developed a multifunctional hydrogel based on CNF@LM particles. While achieving stress–strain and temperature responses, it also has self-healing and antibacterial properties, making it suitable for continuous sensing in extreme environments [135]. Xu et al. fabricated a stretchable, semi-transparent PDMS/LM composite mesh, which forms a conductive network to achieve strain-enhanced electromagnetic shielding and sensing performance [136]. The sensor exhibits anisotropic response and directional recognition capabilities, along with Joule heating stability, making it suitable for multi-modal wearable sensing. Ma et al. developed a self-sensing soft actuator based on an LM @A-MXene composite hydrogel, featuring high conductivity, a wide strain range (≈610%), and excellent sensitivity (GF = 8.92). The device enables light-controlled actuation and re-al-time state monitoring, applicable to multimodal intelligent sensing and soft robotics [137]. Zhou et al. created a hydrogel composite integrating LM with multiple conductive components, achieving dual-mode sensing for both tensile and compressive strains (GF = 9.59 and 33.43, respectively), with rapid response (190 ms) and outstanding durability (1500 cycles), supporting high-precision handwriting recognition and human–machine interaction [138].

In summary, LM-based self-powered sensors are rapidly developing along three major directions: first, the deep integration of multiple energy harvesting modes with sensing functions; second, improving the stability and environmental adaptability of self-powered systems through material and structural innovations; and third, integrating sensing, energy supply, and even driving functions into a single device to build truly energy-independent intelligent systems [139]. In the future, with improved biocompatibility, standardized manufacturing processes, and integration with intelligent algorithms, these sensors are expected to play a vital role in areas such as long-term health monitoring, soft robotics, and the Internet of Things [140].

To provide a unified view of material and electrical performance metrics, we summarize representative LM-based sensors in four major categories: strain, pressure, chemical and multimodal sensing. Their key parameters, including stretchability, resolution, sensitivity, detection range, and durability, are listed in Table 3, together with their typical application scenarios. This overview highlights the advantages of LM sensors in achieving high stretchability, fast response, and stable operation, which are crucial for next-generation wearable devices, health monitoring systems, and soft robotics.

## 5. Liquid Metal in Biomedical Applications

Based on the aforementioned advantages, LMs have achieved breakthroughs in several key fields. Particularly in the biomedical domain, LMs have emerged as core materials for constructing high-performance neural interfaces, artificial vision systems, flexible wearable devices, and multifunctional therapeutic platforms, owing to their excellent biocompatibility, deformability, and multimodal integration capabilities. For instance, flexible neural probes fabricated from LMs can accurately acquire electroencephalogram signals and enable neuromodulation; artificial retina systems integrating LMs with photosensitive arrays have demonstrated significant therapeutic effects in visual function restoration. Moreover, bioelectronic tattoos, ultrasound monitoring electrodes, and photothermal antibacterial patches incorporating LMs have been widely applied in health monitoring, cancer screening, and wound repair, ushering in a new paradigm of intelligent healthcare that integrates “sensing-feedback-intervention”. Beyond biomedicine, LMs also play a pivotal role in flexible electronics and intelligent manufacturing. Through processing techniques such as 3D printing, microfluidic injection, and laser patterning, LMs can construct multi-dimensional deformable circuits, miniaturized sensing networks, electronic skins, and other device architectures, facilitating the development of next-generation “soft robots” and “human-like perception systems”. Furthermore, the integration of LMs with various polymers, hydrogels, nanomaterials, and other composites further enriches their functional properties and application scenarios, including stretchable conductors, self-healing electrodes, and multi-responsive platforms, thereby driving the evolution of materials science from structural-functional integration to adaptive intelligent systems.

Despite their promising application prospects, LMs still face several challenges in practical use, such as unstable interfacial contact caused by uneven oxide layers, insufficient compatibility with certain substrate materials, the need for in-depth verification of long-term in vivo biosafety, limitations in macroscopic structural processing precision, and integration bottlenecks in high-throughput manufacturing. To address these issues, researchers are actively exploring multi-dimensional solutions, including interfacial modification strategies, composite material design, micro-nano processing optimization, biodegradability research, and multi-scale mechanical modeling, to enhance the stability, safety, and industrial feasibility of LM systems.

### 5.1. Neural Signal Monitoring and Visual Function Recovery

In the fields of neural signal monitoring and visual function restoration, LM materials are gradually becoming key components for next-generation neural interfaces and artificial vision systems due to their unique physical properties and excellent biocompatibility. LMs exhibit low modulus, high electrical conductivity, self-healing capability, and excellent formability in flexible electronic devices. These features effectively address the mechanical mismatch issues between traditional rigid metal electrodes and biological tissues, significantly enhancing the accuracy of neural signal acquisition and stimulation.

**Figure 6:**
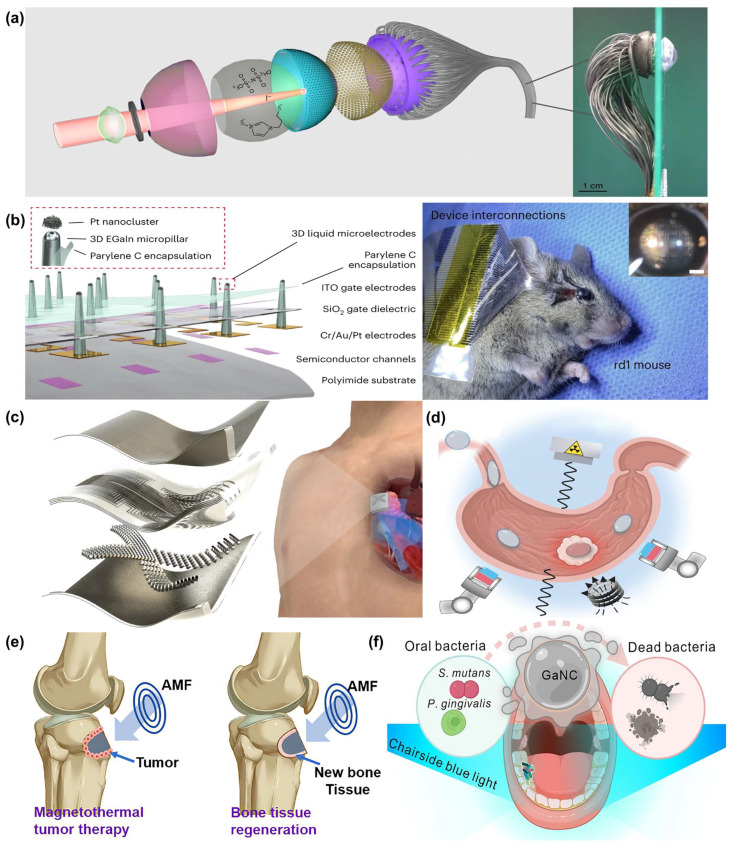
Schematic diagram of LM materials in biomedical applications. (**a**): The detailed structure of a spherical biomimetic electrochemical eye [141]; (**b**): The soft artificial retina with 3D LM microelectrode arrays [142]; (**c**): The wearable cardiac imager with its design and characterization [143]; (**d**): The remote theragnostic gastrointestinal operation using hydrogel-coated containment capsule of magnetic LMs [20]; (**e**): The injectable BPC as bone defect implant materials for osteosarcoma treatment [144]; (**f**): The antibacterial effects of GaNC on oral bacteria under chairside BL [145].

Gu et al. proposed a biomimetic electrochemical artificial eye that utilizes high-density perovskite nanowires to construct a retina and introduces EGaIn conductors to mimic the optic nerve, achieving highly biomimetic visual functionality (Figure 6a). The LM forms conductive connections through flexible tubing, which helps prevent mechanical damage and reduces optical loss. The conductor enables pixel-wise signal readout with high sensitivity and rapid response (19.2 ms), and can detect as few as 86 photons under low-light conditions. The system achieves image reconstruction over a 100.1° field of view, demonstrating the significant potential of LMs in the development of high-performance bio-visual systems [141].

As shown in Figure 6b, Chung et al. developed a flexible artificial retina system by integrating a photosensitive transistor array with EGaIn-based 3D printed stimulation electrodes, enabling a safe and minimally invasive epiretinal implantation [1]. The LM’s low modulus, strong biocompatibility, and excellent formability allow the electrodes to conform intimately to the retinal surface, significantly reducing tissue damage. Compared with traditional rigid electrodes, LM electrodes effectively deliver electrical charges to retinal neurons, enhancing stimulation efficiency. In experiments, localized light stimulation successfully activated the retinal ganglion cells of degenerated retinal mouse models, and machine learning analysis of the evoked signals confirmed restored visual function [142].

Park et al. developed a soft neural probe based on EGaIn and a monolithically integrated system with cranial electronic devices for high-resolution neuronal activity recording [46]. The probe has a diameter of only 5 μm and exhibits softness and low invasiveness comparable to that of neurons, enabling precise implantation into various brain regions. The use of LMs provides excellent electrical conductivity, self-healing capability, and deformability, effectively preventing signal interruption.

Zhang et al. developed a 20-channel neural electrode array based on LMs, encapsulated with platinum, exhibiting excellent flexibility and conformability for close adherence to the skull, thereby overcoming the mechanical mismatch between traditional rigid electrodes and biological tissues. In vivo experiments demonstrated that the LM electrodes successfully recorded electroencephalogram signals from rats under low flow or deep anesthesia and detected auditory evoked potentials triggered by sound stimuli. With the aid of source localization techniques, the activated brain regions were precisely identified [146].

Zhang et al. also developed a hawk-claw-inspired LM-encapsulated microneedle patch to promote incisional wound healing. Fabricated by a multi-mold guided photolithography method, the patch consists of two inclined hydrogel microneedle sections connected by breathable gauze, forming a clamping structure that closely adheres to the skin and stabilizes the wound area. LM is embedded in a W-shaped pattern within the microneedles, connecting the needle tips to an external power source to generate a stable spatial electric field, which enhances cell migration and tissue regeneration. Animal experiments confirmed that the patch significantly accelerated wound healing in a Sprague–Dawley rat model [147].

### 5.2. Wearable Devices and Biological Signal Monitoring

LM-based wearable devices and biological signal monitoring systems are increasingly emerging as a vital direction in the convergence of flexible electronics, biomedicine, and human–machine interaction. Owing to their unique physicochemical properties, LMs demonstrate outstanding advantages in the development of soft, skin-conformal, and functionally integrated wearable systems. Current research focuses on composite strategies involving LMs with polymers, nanomaterials, and hydrogels to construct sensing platforms with self-healing, reconfigurable, highly stretchable, and miniaturized characteristics. These platforms have successfully enabled real-time, high-precision monitoring of various physiological signals, including body temperature, electrocardiogram (ECG), acoustic signals, and ultrasound imaging.

Shi et al. developed a multifunctional wearable electronic system by integrating EGaIn with dynamic polyimine, exhibiting excellent conductivity, mechanical stretchability, and self-healing capability, enabling multimodal monitoring of body temperature, ECG, and acoustic signals [148]. Upon mechanical damage, the LM flows to refill the fractured regions, while the dynamic bond exchange within the polyimine network facilitates circuit self-repair. Lim et al. fabricated a stretchable electronic textile by introducing a gold nanoparticle encapsulation layer onto the surface of LM, achieving biocompatibility, stable conductivity, and strong adhesion. The textile demonstrated outstanding electrochemical and bioelectrical performance in sweat analysis and physiological signal monitoring during physical activity [36]. Lee et al. designed a composite electronic tattoo composed of LM particles and a platinum-decorated carbon nanotube coating, which can be brush-applied onto the skin within 10 s. The tattoo offers high conductivity, biocompatibility, and pattern resolution, making it suitable for applications such as ECG monitoring and photothermal therapy [149].

As shown in Figure 6c, Hu et al. developed a high-density multilayer stretchable electrode based on an EGaIn-SEBS composite for wearable ultrasound imaging devices, enabling continuous, real-time, and noninvasive cardiac function monitoring. EGaIn imparts excellent conductivity and patternability to the device. The electrode exhibits approximately 110% stretchability and a Young’s modulus of only 921 kPa, matching the mechanical properties of human skin to ensure stable adhesion. The interfacial bonding strength reaches 236–250 kPa, significantly surpassing that of conventional adhesives, while also providing superior electromagnetic shielding to improve the signal-to-noise ratio. Combined with a deep learning model, the system can automatically extract metrics such as left ventricular ejection fraction and cardiac output. This work demonstrates the critical role of LM in high-performance, flexible cardiac monitoring systems and offers a new path for intelligent cardiovascular health management [143].

Fang et al. developed a self-healing bioelectronic patch integrated with EGaIn for real-time dynamic monitoring of methylated ctDNA, aiming to facilitate early cancer diagnosis. The EGaIn microfluidic circuit, fabricated via projection micro-stereolithography, exhibits a resolution of 10 μm, maintains conductivity under 100% strain, and possesses automatic self-healing capability after damage. The patch integrates transdermal interstitial fluid sampling with a FET biosensor, enabling sensitive detection of ctDNA concentrations as low as 10^−16^ M and supporting phenotypic analysis in small animal tumor models. In this platform, the LM offers high conductivity and mechanical adaptability, significantly enhancing functional integration and detection accuracy in wearable devices[150]. Figure 6d illustrates the magnetically controlled LM capsule system developed by Shen et al. for remote gastrointestinal operations. The core of the system is magnetic LM, which features high electrical conductivity (~3.4 × 10^4^ S/cm) and magnetic responsiveness, encapsulated in a high-strength double-layer hydrogel shell. Under a 5 mT magnetic field, the structure enables remote navigation and operation at a velocity of 7.5 mm/s, while maintaining mechanical integrity under pressure up to 350 kPa, suitable for the moist and peristaltic gastrointestinal environment. The deformability of the LM allows the capsule to precisely deploy in narrow spaces and perform tasks such as localized drug release and electrical stimulation [20].

### 5.3. Tumor and Systemic Disease Treatment

In the field of tumor therapy and systemic disease intervention, LMs are emerging as core materials for next-generation intelligent therapeutic platforms, owing to their excellent photothermal responsiveness, electromagnetic regulation capability, biocompatibility, and tunable functionality. They have demonstrated significant advantages in photothermal therapy (PTT), magnetothermal therapy (MTT), combined chemo-/immunotherapy, and tissue regeneration.

As shown in Figure 6e, Wang et al. developed an injectable bismuth-based composite (BPC) for bone defect repair and osteosarcoma treatment [144]. By compounding low-melting-point BiInSn alloy with clinically used PMMA bone cement, the resulting BPC material exhibits excellent injectability, formability, and significantly enhanced mechanical strength (compressive strength increased by ~252%). Under an alternating magnetic field, the BPC material generates mild heating (43–45 °C) through magnetothermal effects, effectively killing tumor cells. Simultaneously, this mild thermal stimulation promotes new bone formation, with both in vitro and in vivo experiments confirming enhanced osteogenic activity. Toxicological analysis verified good hepatic and renal function and cell viability, indicating excellent biocompatibility and application potential.

Shen et al. encapsulated LM within a ZIF-8 framework and loaded curcumin and doxorubicin to construct an LM-based nanodrug delivery system with cascade-controlled release under acidic conditions and near-infrared (NIR) irradiation [13,15]. This system exhibited excellent photothermal conversion and biocompatibility, effectively targeting tumor bone metastases, inhibiting transforming growth factor-β expression and extracellular matrix remodeling, and alleviating immunosuppressive environments, demonstrating the great potential of LM in precise tumor immunotherapy. Hou et al. developed an LM-based multimodal tumor therapy platform that integrates the high thermal conductivity and softness of GaIn-Cu composite paste to achieve cryoablation-induced cooling and analgesia for skin tumors. Subsequently, photothermal therapy using Pluronic F127-modified LM nanoparticles significantly enhanced the therapeutic effect, enabling non-invasive melanoma ablation [151].

Low-melting-point LM gallium exhibits biocompatibility, excellent electrical and thermal conductivity, and dual functionalities of antibacterial activity and bone regeneration. Liu et al. proposed a Ga-based nanocomposite for multifunctional treatment of periodontitis (Figure 6f) [145]. Ga^3+^ ions released from LM inhibit pathogenic bacterial metabolism via a “Trojan horse” mechanism, while graphene quantum dots and blue light irradiation achieve multimodal antibacterial effects. In vitro and in vivo studies demonstrated significant inhibition of Porphyromonas gingivalis infection, regulation of bone homeostasis, promotion of osteogenesis, and suppression of osteoclastogenesis, highlighting LM’s dual regulatory role in antibacterial therapy and bone repair.

Beyond antibacterial and bone-regeneration effects, the biocompatibility and safety evaluation of LMs has also attracted increasing attention in biomedical engineering. Owing to their fluidity, flexibility, and low melting points, Ga- and Bi-based LMs can conform closely to soft biological tissues, thereby minimizing mechanical stress concentration and inflammatory responses. This physical adaptability not only prolongs implant lifespan but also supports the development of long-term stable implantable electronic devices [71].

Recent studies have reported that LMs exhibit excellent shape compliance and phase-transition behavior in vivo. For example, injected LM can deform to match tortuous blood vessels and subsequently solidify to achieve vascular embolization, providing a minimally invasive and safe strategy for aneurysm or tumor treatment [152]. Similarly, in bone repair, Bi-based LMPMs demonstrate modulus matching with surrounding tissues, enabling defect filling without thermal damage and maintaining integration for over 200 days in animal studies [153]. These findings highlight the safety indicators of LM implants, including stable phase transition near physiological temperature, elastic modulus compatibility, and long-term tissue integration.

Li et al. developed a 3D-printed LM-based antibacterial hydrogel scaffold for wound healing. Micron-sized LM droplets were generated in a gelatin solution via ultrasonic treatment, imparting the scaffold with good temperature sensitivity and printability. The incorporation of LM enabled photothermal responsiveness, allowing efficient conversion of light into heat for antibacterial action. Additionally, the scaffold encapsulated vascular endothelial growth factor, which was released upon heating. Animal experiments demonstrated effective bacterial clearance, enhanced angiogenesis, and accelerated wound closure [73]. Nguyen et al. developed a bioactive coating based on silver-gallium LM particles. The LM imparts excellent broad-spectrum antibacterial properties, effectively inhibiting Pseudomonas aeruginosa, Staphylococcus aureus, and their drug-resistant variants. The antibacterial mechanisms include reactive oxygen species generation, cell membrane disruption and disruption of key bacterial metabolic pathways [154].

He et al. proposed an injectable bismuth-based LM alloy for long-term bone repair, demonstrating its multifunctionality in orthopedic biomedicine. The LM remains plastic at body temperature with a melting point of 61.5 °C, and rapidly solidifies into a stable scaffold with a strength of 50 MPa, close to cortical bone. Under a magnetic field, it shows remote magnetothermal effect, reaching 47 °C in 20 s to assist sterilization and inhibit tumor recurrence. It also exhibits good bone affinity, increasing the bone bonding area and promoting osteogenic marker expression [153]. Guan et al. developed composite nanoparticles based on EGaIn and p-phenylenediamine (PPD), featuring antibacterial edge structures and good thermal responsiveness. Applied in skin patches, the nanoparticles enable electrochemical detection of C-reactive protein with a sensitivity of 0.5 mg/L, suitable for inflammation monitoring. The photothermal patch exhibits 83% photothermal conversion efficiency, effectively achieving anti-inflammatory effects and wound healing [128].

LMs, with their material properties of highly integrated structure and function, are undergoing a strategic transformation from single-point treatment to whole-course intervention. In the future, by integrating multimodal imaging technologies, responsive drug design, and AI-driven precise regulation, LMs are expected to play a more significant role in personalized tumor therapy, chronic disease management, regenerative medicine, and other fields, serving as a crucial driving force for technological innovation in systemic disease intervention.

## 6. Conclusions and Future Perspectives

LMs have emerged as a transformative class of functional materials that integrate fluidic mechanics with metallic conductivity, offering remarkable advantages in terms of mechanical compliance, interfacial adaptability, and dynamic reconfigurability. Over the past decade, research into LMs has advanced from fundamental studies of their physical and chemical behaviors to increasingly diversified applications in additive manufacturing, electrocatalysis, flexible sensing, and biomedical systems. This review has comprehensively examined the representative advances in four major domains: LM-based 3D printing, catalysis, sensing, and biomedical applications, illustrating the unique roles LMs play across multiscale and multiphysics contexts.

In 3D printing, LMs have demonstrated unparalleled printability, enabling direct writing of conductive paths, antennas, and stretchable electronics with micron-scale precision [155]. Their ability to maintain structural integrity under deformation has led to the fabrication of robust soft circuits and reconfigurable devices [55]. Moreover, hybrid strategies involving encapsulation, emulsification, and rheological tuning have further expanded the compatibility of LMs with various substrates and printing platforms, paving the way for intelligent, multifunctional systems. In catalysis, the dynamic surface of LMs, free of fixed lattice constraints, allows for continuous atomic reconfiguration, supporting the formation of highly active and self-renewing catalytic sites. Studies have shown that LMs can serve as both catalyst hosts and electron reservoirs, enabling tunable reaction pathways, especially in CO_2_ reduction, ammonia synthesis, hydrocarbon oxidation, and waste plastic depolymerization. By doping with noble or transition metals and leveraging the electron spillover effect, LM-based catalysts have achieved enhanced reaction selectivity, improved resistance to poisoning, and prolonged operational stability. Notably, their application in green energy conversion [87] and environmental remediation [93] holds immense promise. In the sensing field, LMs offer a powerful combination of high conductivity, softness, and deformability. This makes them ideal candidates for strain, pressure, and multimodal sensors that require intimate skin or substrate conformity. The development of LM-based capacitive, resistive, magnetic, and inductive sensors has enabled real-time monitoring of complex mechanical stimuli with high spatial and temporal resolution. Their compatibility with machine learning algorithms for signal decoupling and pattern recognition further augments their application in human–machine interaction and soft robotics [49]. Biomedical applications of LMs have also witnessed rapid progress. Their intrinsic biocompatibility, low Young’s modulus, and high conformability to soft tissues have facilitated the design of next-generation wearable and implantable devices. From self-healing electronic skins to stretchable ultrasound patches and bioelectronic tattoos, LM-enabled platforms have demonstrated superior functionality in long-term physiological monitoring, targeted therapy, and non-invasive diagnostics. The combination of LM-based materials with hydrogels, nanofibers, and bioresponsive systems has further enhanced their utility in personalized healthcare.

Despite these achievements, challenges remain. The oxidation, leakage, and long-term stability of LM components require attention for clinical and industrial translation [156]. Advanced encapsulation techniques, surface chemistry modulation, and alloy engineering are necessary to overcome these issues. Furthermore, scalable fabrication strategies and environmentally friendly processing routes will be critical for the widespread adoption of LM technologies. Interdisciplinary collaboration—combining materials science, biomedical engineering, electronics, and AI—is expected to play a vital role in driving the next wave of LM-based innovations. In conclusion, LMs represent a unique and versatile material system with cross-domain impact. Their ability to bridge soft and rigid systems, adapt dynamically to complex environments, and integrate multifunctional performance offers an exciting frontier for future research and application. With continuous breakthroughs in material design, device integration, and theoretical understanding, LM-based technologies are poised to make profound contributions to the advancement of intelligent manufacturing, sustainable catalysis, adaptive sensing, and precision medicine.

## Figures and Tables

**Table 1:** The physical properties of prominent LMs [26,27,28].

LM	Melting Point [°C]	Density [10^3^ kg m^−3^]	Electrical Conductivity [10^6^ S m^−1^]	Thermal Conductivity [W m^−1^ K^−1^]
Ga	29.76	5.91 (at 25 °C)	3.7	29.3
EGaIn	15.00	6.28 (at 20 °C)	3.4	26.6
Galinstan	−19.00	6.44 (at 20 °C)	3.5	16.5
BiIn_48.8_Sn_19.6_	60.20	8.04	1.9	14.5

**Table 2:** Comparison of representative LM 3D printing techniques [43].

Printing Technology	Advantages	Limitations	Technical Parameters	Applications
Direct-Ink Writing	High precision; simple control	Poor stability; easily oxidized	Moderate printing speed	Neural probes; interconnects
Embedded Printing	High stretchability	low throughput	Slow printing speed; high shape fidelity	Bioelectronics; morphing systems
Extrusion and Infiltration Printing	Excellent material compatibility	Lower resolution; non-reusable	Fast printing speed	Thermal regulators; microelectronics

**Table 3:** Representative performance indicators of LM-based strain sensors.

Sensor Type	Stretchability	Gauge Factor (GF)	Conductivity/ Stability	Applications	Ref.
LM hydrogel (SA-modified)	382%	3.66	Conductive at −40 °C	Low-temperature wearable electronics	[117]
LM/CNT/PDMS ternary composite	50–100%	5.35	Low hysteresis <5%	Continuous wearable monitoring	[115]
LM/Ni magnetic particle composite	300%	5.17	<8% after 2000 cycles	Flexible human–machine interfaces	[116]

**Table 4:** Representative performance indicators of LM-based pressure sensors.

Sensor Type	Stretchability	Sensitivity	Response Speed	Applications	Ref.
LM microelectrode sensing array	Flexible	7.42 kPa^−1^	54 ms	Pressure mapping	[121]
LM-carbon nanofiber foam strain sensor	Lightweight flexible	3.8 kPa^−1^	83 ms	Physiological signal detection	[123]
Tunable dual-mode LM pressure sensor	Adaptive	Soft mode: 16.97 kPa^−1^; Rigid mode: up to 1.45 MPa	/	wide-range pressure sensing	[119]

## Data Availability

Data sharing is not applicable.

## Abbreviations

The following abbreviations are used in this manuscript:

ABS	Acrylonitrile butadiene styrene
AI-LMS	Anisotropic inductive liquid metal sensor
BPC	Bismuth-based composite
CNF	Cellulose nanofibers
CuGa-LM	CuGa2-loaded liquid metal
Cu-LMC	Cu-doped liquid metal catalyst
DIW	Direct ink writing
ECG	Electrocardiogram
FDM	Fused deposition modeling
GF	Gauge factor
LMs	Liquid metals
LMS	Liquid metal silicon
LMPs	Liquid metal microparticles
MTT	Magnetothermal therapy
NIR	Near-infrared
NO	Nitric oxide
PMA	Polymethyl methacrylate
PPT	P-phenylenediamine
PTT	Photothermal therapy
PVC	Polyvinyl chloride
rGO	Reduced graphene oxide
RH	Relative humidity
Ru	Ruthenium
SCALMS	Supported catalytically active liquid metal solution
SNR	Signal to noise ratio
TENG	Triboelectric nanogeneration
TPMS	Triply periodic minimal surface
VOC	Volatile organic compound
3D	Three-dimensional
3e-3DP	Embedded 3D printing
